# Effect of pH on Diclofenac–Lysozyme Interaction: Structural and Functional Aspect

**DOI:** 10.3389/fmolb.2022.872905

**Published:** 2022-07-11

**Authors:** Mohd Basheeruddin, Sheeza Khan, Neesar Ahmed, Shazia Jamal

**Affiliations:** School of Life Sciences, B. S. Abdur Rahman Crescent Institute of Science and Technology, Chennai, India

**Keywords:** diclofenac sodium, lysozyme, thermal denaturation, protein stability, circula dichroism spectrum

## Abstract

As a nonsteroidal antiinflammatory drug, diclofenac (DCF) is used in the treatment of a variety of human ailments. It has already been reported that the use of this class of drugs for a longer duration is associated with numerous side effects such as cardiovascular implications, reno-medullary complications, etc. In the present study, the effect of DCF on the structure, stability, and function of lysozyme was studied. The study was designed to examine the effect of DCF only at various pH values. Heat-induced denaturation of lysozyme was analyzed in the presence and absence of various molar concentrations of DCF at different pH values. The values of thermodynamic parameters, the midpoint of denaturation (*T*_m_), enthalpy change at *T*_m_ (Δ*H*_m_), constant pressure heat capacity change (Δ*C*_p_), and Gibbs energy change at 25°C (Δ*G*_D_^o^), thus obtained under a given set of conditions (pH and molar concentration of DCF), demonstrated the following 1) DCF destabilized lysozyme with respect of *T*_m_ and Δ*G*_D_^o^ at all the pH values, 2) the magnitude of protein destabilization is lesser at acidic pH than at physiological pH, 3) structural changes in lysozyme are less projecting at pH 2.0 than at pH 7.0, and 4) quenching is observed at both pH values. Furthermore, the process of protein destabilization in the presence of DCF is entropically driven.

## Introduction

Protein–drug interaction studies are important and central in understanding biological processes. Such interactions may influence the transportation, absorption, metabolism, and excretion of drugs (Caldwell et al., 1995). Small ligands are known to intermingle with these molecules readily (Ajmal et al., 2017a; Zhang et al., 2020). Recently, such studies are hot spots of multidisciplinary research (Ajmal et al., 2017b; Zhang et al., 2020). Proteins are versatile molecules and perform many different functions in the human body. They are flexible molecules, and ligand binding can affect their hydrodynamics and function; these alterations can be harmful or useful (Babu et al., 2011; Elfaki et al., 2013; Ajmal et al., 2017a). Drug binding to transport proteins can significantly affect the metabolism of drug molecules. It becomes important to look at the different aspects of these interactions when designing the dosage of the drugs spatially in a multidrug therapy or treatment in comorbid conditions, where the picture can be more complicated; protein binding of drugs not only affects drug pharmacokinetics but can also affect its function.

Diclofenac (DCF) sodium and potassium salts have been used to treat a range of ailments including osteoarthritis, ankylosing spondylitis, rheumatoid arthritis, primary dysmenorrhea, and mild to moderate pain (Sharma et al., 2012; Tampucci et al., 2019). DCF is a nonsteroidal antiinflammatory drug that is a derivative of phenylacetic acid; that is, its chemical name is 2-(2,6-dichloroanilino) phenylacetic acid (Vane and Botting, 1996; Ibrahim et al., 2018; Boumya et al., 2021; Galisteo et al., 2021). It is an analgesic, antipyretic, and antirheumatic medicament. DCF use has also been implicated in defective cardiovascular function. Numerous studies exist implicating the role of DCF in cardiac, renal, and gastrointestinal complications (Gökçimen et al., 2000; Weir, 2002; Lewis et al., 2002; Baigent et al., 2013; Lundgren et al., 2017). Lysozyme is a small globular protein used as a model molecule to study the effect of external agents on its stability and functions (Ajmal et al., 2017b; Leone et al., 2019). Ever since its discovery, lysozyme has represented a prototype molecule for understanding the complexity of its structure and function (Saadati-Eskandari et al., 2019). Thus, the study on the interaction of drugs with lysozyme has important significance. Such studies are useful for providing information on the structural features of the molecule interaction with drugs and illuminating the therapeutic effectiveness of drugs (Ajmal et al., 2016; Karaman and Sippl, 2019). Interestingly, no study exists to date that could explain the pH dependence of DCF effects on the structural, functional, and stabilization properties of proteins. In this communication, we have analyzed the effect of DCF on the structure, stability, and function of lysozyme at different pH values by measuring ∆*G*_D_^o^ (Gibbs free energy change upon denaturation at 25°C) and enzyme kinetic parameters (*K*_m_ and *k*_cat_) in the presence and absence of DCF.

## Materials and Methods

Lyophilized hen egg-white lysozyme and *M. luteus* cell wall were commercially available and purchased from Sigma. The ultrapure sample of guanidinium chloride (GdmCl), DCF, cacodylate, sodium acetate, and dialysis tubing was also procured from Sigma. KCl and *glycine* were obtained from SRL. All analytical grade chemicals were used without any further purification.

The stock solution of lysozyme was immensely dialyzed against 0.1 M KCl at pH 7.0. This solution was filtered with 0.45 μm millipore filter paper. Molar absorption coefficient (M^−1^ cm^−1^) values of 39,000 at 280 nm for lysozyme were used to determine the concentration of protein (Sinha et al., 2000; Lindorff-Larsen, 2019). Refractive index measurements were used to find the concentration of GdmCl stock solution. All solutions were prepared in an appropriate buffer that contains 0.1 M KCl. In this study, 50 mM KCl–HCl buffer (pH2.0), 50 mM glycine–HCl buffer (pH3.0), 50 mM sodium acetate buffer (pH 4.0), and 50 mM cacodylic acid buffer (5.0–7.0) were used. The solutions of DCF were prepared in the respective buffers at different pH values. Heating or the addition of GdmCl may cause a change in pH; hence, the pH of the samples was measured before and after the experiment. There were no such changes observed at all pH values. All the solutions used were prepared fresh each time.

Heat-induced denaturation experimental studies were carried out in a spectrophotometer (Jasco Model: V-730 UV/VIS) with a temperature controller (peltier Model ETCS-761). At the rate of 1°C/min, the samples were heated, and this scan rate provides sufficient time for equilibration. All samples were thermally denatured in the temperature range of 20°C–85°C. An increase in temperature shows the variation in absorbance at 300 nm. Total data points were collected after thermally denaturing the samples. At a given wavelength, the absorbance values were converted to Δ*ε*_λ_(M^−1^ cm^−1^), the difference molar absorption coefficient. All heat-induced transition curves were plotted as Δ*ε* versus temperature. *T*_m_ and Δ*H*_m_ were determined from these plots using Eq. 1 (Santoro and Bolen, 1988; Sinha et al., 2000; Singh et al., 2007; Lin et al., 2020; Leite et al., 2021).

$$y\left(T\right)=\frac{y_{N}\left(T\right)+y_{D}\left(T\right)\exp\left[-\text{Δ}H_{m}/R\left(1/T-1/T_{m}\right)\right]}{1+\exp\left[-\text{Δ}H_{m}/R\left(1/T-1/T_{m}\right)\right]}$$ (1)

The parabolic function (such as *y*_N_(*T*) and *y*_D_(*T*)) for the analysis of the transition curve explains the dependence of the optical properties of the folded and unfolded protein molecules (Islam, 2020; Parray et al., 2020; Parray et al., 2021). The value of temperature-independent Δ*C*_p_ was determined by using the slope of the plot between Δ*H*_m_ and *T*_m_ using Eq. 2 (Becktel and Schellman, 1987; Sinha et al., 2000; Rahman et al., 2015; Dragan et al., 2019).

$$\text{Δ}C_{\rho }=\left(\partial \text{Δ}H_{m}/\partial T_{m}\right)$$ (2)

With the values of *T*_m_, Δ*H*_m_, Δ*C*_p_, and Δ*G*_D_(*T*), the values of Δ*G*_D_ were estimated at any temperature using the Gibbs–Helmholtz equation (Eq. 3) (Singh et al., 2005; Khan et al., 2013; Dragan et al., 2019; Naiyer et al., 2021).

$$\text{Δ}G_{D}\left(T\right)=\text{Δ}H_{m}\left(\frac{T_{m}-T}{T_{m}}\right)-\text{Δ}C_{p}\left[\left(T_{m}-T\right)+T\text{ln}\left(\frac{T}{T_{m}}\right)\right]$$ (3)

The far-UV CD of lysozyme was measured in a Jasco spectropolarimeter (Model: J-810) having a temperature controller (peltier model-Jasco PTC-424S). The cuvette path length used for far UV was 1 mm. At each wavelength, the value of mean residue ellipticity (deg cm^2^ dmol^−1^) was converted by the CD signal using Eq. 4.

$${\left[\theta \right]}_{\lambda }=\theta_{\lambda }M_{0}/10lc$$ (4)

Where the observed ellipticity in milli degrees is *θ*_λ_ at wavelength λ_nm_, M_0_ is the mean residue weight of the protein, c is the protein concentration in mg cm^−3^, and *l* is the path length (cm).

Fluorescence studies were carried out at different concentrations of DCF (2–20 µM) at two pH values (i.e., 2.0 and 7.0). Fluorescence quenching was monitored by measuring intrinsic fluorescence from the range of 315–500 nm with the excitation wavelength of 295 nm. The slits were set at 5 nm for the excitation and emission.

The *M. luteus* cell wall was used as a substrate for the lytic activity of lysozyme at pH 7.0 at 25°C. The effect of different concentrations of DCF on kinetic parameters (*K*_m_ and *k*_cat_) was measured using the method of Maurel and Douzou (1976). The given concentrations of DCF were preincubated with the substrate and the enzyme. The change in absorbance on the addition of lysozyme to the substrate with constant stirring was recorded at 450 nm in a spectrophotometer (Model: Jasco V-660 UV/Visible). The slope of the linear part (the first 30 s) was considered to find out the rate of lysis, as in this region 10–20% of the substrate was lysed. The value of apparent specific absorbance (*ε*_450_) of the *M. luteus* cell wall was taken as 0.656 mg/L (Khan et al., 2013). The weight of cells lysed per second per mol of lysozyme is defined as the rate of lysis of lysozyme. The substrate was directly taken in a glass cell with a 1 cm path length with concentrations ranging from 10 to 200 mg m1^−1^. The final volume of solutions was made to 3 ml with buffer. Readings were taken in the spectrophotometer at 25°C ± 0.1°C. A constant amount of lysozyme (0.45 mM) was added to initiate the reaction in all samples. The kinetic parameters *K*_m_ and *V*_max_ were calculated from Michaelis–Menten plots (Eq. 5),

$$v=V_{max}\left[S\right]/\left(K_{m}+\left[S\right]\right)$$ (5)

where, the initial velocity is *v*, and the concentrations of the substrate are [S]. The product of enzyme concentration and *V*_max_ gives the value of *k*_cat_.

## Results and Discussion

**FIGURE 1:**
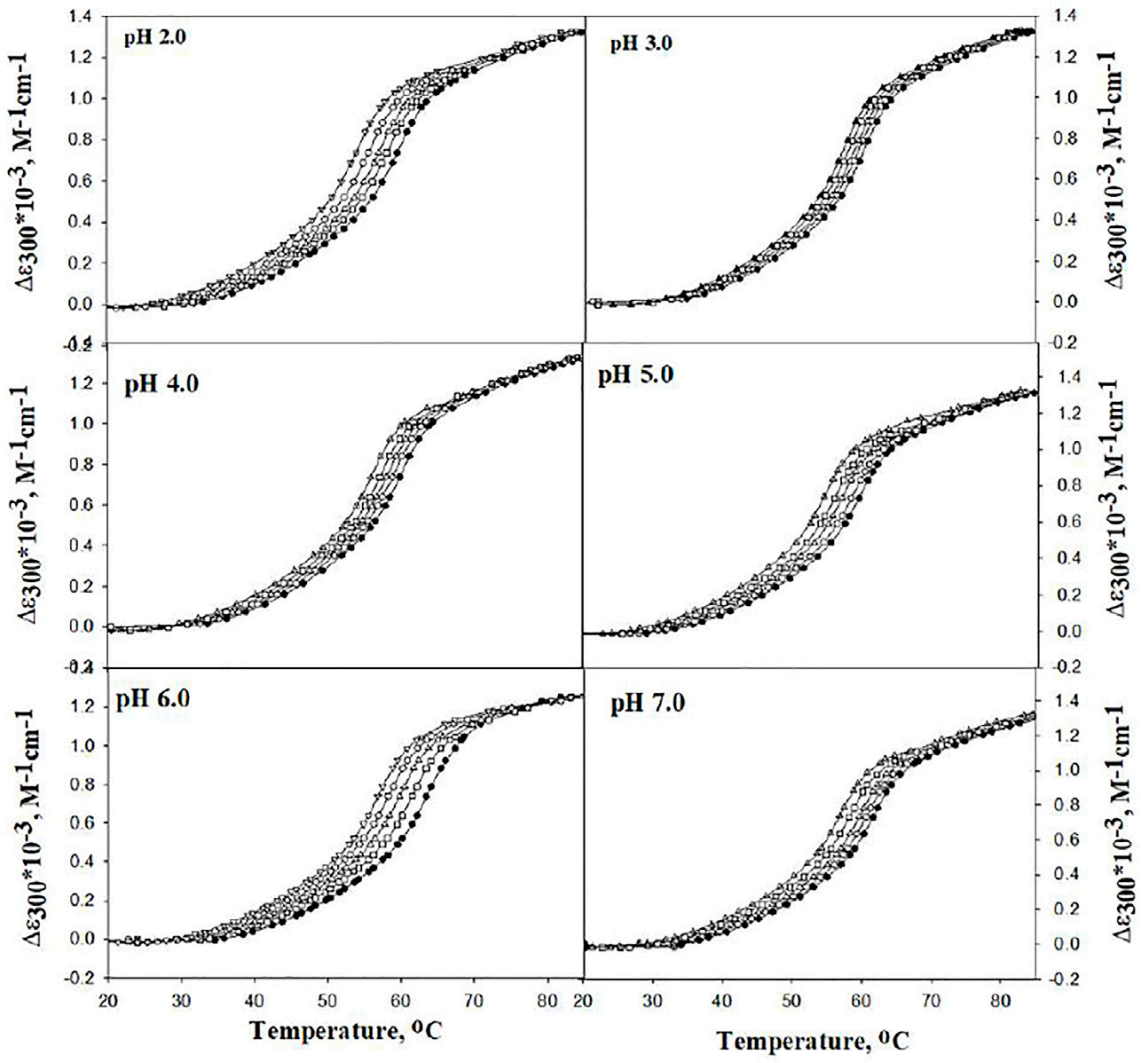
Representative thermal denaturation curves of lysozyme in the absence and presence of different concentrations of DCF at different pH values. Symbols in figures: (●), (□), (∆), (○), and (▽) represent 0, 5, 10, 15, and 20 μM of DCF, respectively.

The effect of DCF on the stability of lysozyme was investigated by measuring the heat-induced denaturation of lysozyme in the presence of different concentrations of DCF (5–20 µM) at various pH values (i.e., 2.0, 3.0, 4.0, 5.0, 6.0, and 7.0). Figure 1 explains the representative thermal denaturation curves of lysozyme.

The differences in molar absorption coefficient changes were observed in ∆*ɛ*_300_ as a function of temperature. The values for *T*_m_ and Δ*H*_m_ were analyzed using Eq. 1. There was no complete transition in the range from 20°C to 80°C at pH 5.0, 6.0, and 7.0. Hence, 2.0 M GdmCl was added to bring down the denaturation curves in the range that can be measured, and GdmCl effects were corrected using the earlier published method (Becktel and Schellman, 1987; Singh et al., 2005; Khan et al., 2013; Shahid et al., 2019; Chowhan et al., 2021). Table 1 shows the values of *T*_m_ and Δ*H*_m_ at pH 5.0, 6.0, and 7.0 were corrected for the contribution of GdmCl.

**TABLE 1:** Thermodynamic parameters of lysozyme in the presence of different concentrations of DCF at different pH values.^a^ ^,^ ^b^ ^.^

pH	[DCF], μM	*T*_m_	ΔH_m_	Δ*G*_D_^o^
2.0	0	58.0	82	5.63
	5	56.6	84	5.35
	10	54.9	81	5.14
	15	54.0	78	5.05
	20	53.2	77	4.99
3.0	0	77.3	98	8.04
	5	76.5	96	7.89
	10	75.9	97	7.74
	15	75.0	94	7.60
	20	74.2	95	7.45
4.0	0	79.0	102	8.64
	5	78.0	100	8.49
	10	77.2	98	8.34
	15	76.3	101	8.19
	20	75.1	99	7.89
5.0	0	80.0	118	11.05
	5	78.4	117	10.77
	10	77.1	113	10.42
	15	76.0	114	10.08
	20	74.5	112	9.73
6.0	0	84.0	128	12.77
	5	82.0	126	12.31
	10	80.3	122	11.77
	15	78.1	119	11.35
	20	76.7	119	11.15
7.0	0	86.0	128	13.00
	5	83.9	125	12.54
	10	82.1	126	12.16
	15	80.5	124	11.78
	20	78.7	125	11.24

a From triplicate measurements, values of maximum errors from the means are 0.2–0.5, 2–5, and 3–5% in *T*_m_, Δ*H*_m_, and Δ*G*_D_^o^, respectively.

b *T*_m_, Δ*H*_m_, and Δ*G*_D_^o^, are in °*C*, kcal mol^−1^, and kcal mol^−1^, respectively.

It can be seen that *T*_m_ decreases with an increase in the concentration of DCF at all pH values. This can also be observed in the shift of denaturation curves in Figure 1 toward the left side. This decrease in the *T*_m_ is less at pH 2.0. The values of *T*_m_ and Δ*H*_m_ (both at a specific molar concentration of DCF) at different pH values were plotted in a graph, and the slope of the straight line of the *T*_m_ and Δ*H*_m_ (as specific molar concentrations of DCF) at different pH values gives the value of Δ*C*_p_ [i.e., Δ*C*_p_ = (δΔ*H*_m_/δΔ*T*_m_)]. The Δ*C*_p_ values obtained here and those obtained from DSC measurements are in agreement with an earlier report by Makhatadze and Privalov (1993; Eskew and Benight, 2021). However, *T*_m_ is not a good measure of protein stability as the stability (∆*G*_D_^o^) depends not only on *T*_m_ but also on Δ*C*_p_ and Δ*H*_m_. Therefore, we determined the ∆*G*_D_^o^ values at different experimental conditions using Eq. 3. Values of Δ*G*_D_ at 25°C (i.e., Δ*G*_D_^o^) were calculated at all pH values with the help of the *T*_m_, Δ*H*_m_, and Δ*C*_p_ values with Eq. 3. The values for ∆*G*_D_^o^ given in Table 1 shows that an increase in the concentration of DCF decreases the values of ∆*G*_D_^o^ at all pH values and also that the destabilizing effect of DCF is less at pH 2.0 than at pH 7.0.

**FIGURE 2:**
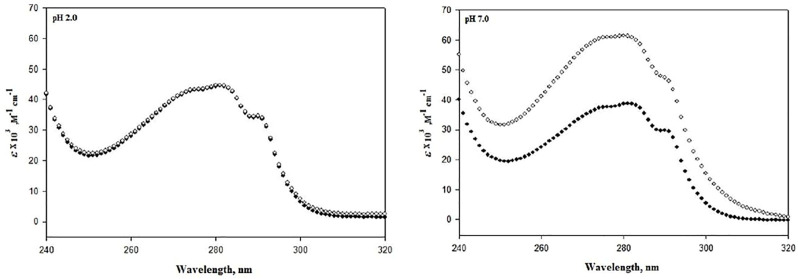
Absorbance spectra of lysozyme in the absence (●) and presence of 20 μM DCF (○) at pH 7.0 and 2.0 at 25°C.

Since structure determines stability, this decrement in instability should also be reflected in the structure of lysozyme; hence, structural studies on lysozyme were carried out. Figure 2 represents the absorption spectra of lysozyme in the absence and presence of the highest concentrations of DCF (20 μM) at pH 7.0 and pH 2.0. Observing the changes in the tertiary structure, it can be seen that pH 2.0 demonstrates no change, while significant change was observed at pH 7.0.

**FIGURE 3:**
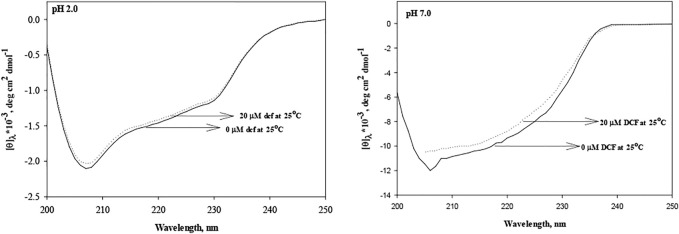
Secondary structures of lysozyme in the absence and presence of DCF at pH 2.0 and 7.0.

Further, to analyze the effect of DCF on the secondary structure of lysozyme, far-UV CD experiments were conducted in the absence and presence of 20 μM DCF. Monitoring the secondary structure probe (222 nm), a significant change can be observed at pH 7.0, but there were no significant changes at pH 2.0 (Figure 3). The changes in the absorption spectra depend on side chains of chromophores, tyrosine, and tryptophan (Wetlaufer, 1963; Pignataro et al., 2020), while far-UV CD demonstrates changes in the peptide backbone conformation. Therefore, it can be concluded that DCF induces loss of structure of lysozyme at pH 7.0, which is also reflected in protein stability in terms of thermodynamic parameters.

**FIGURE 4:**
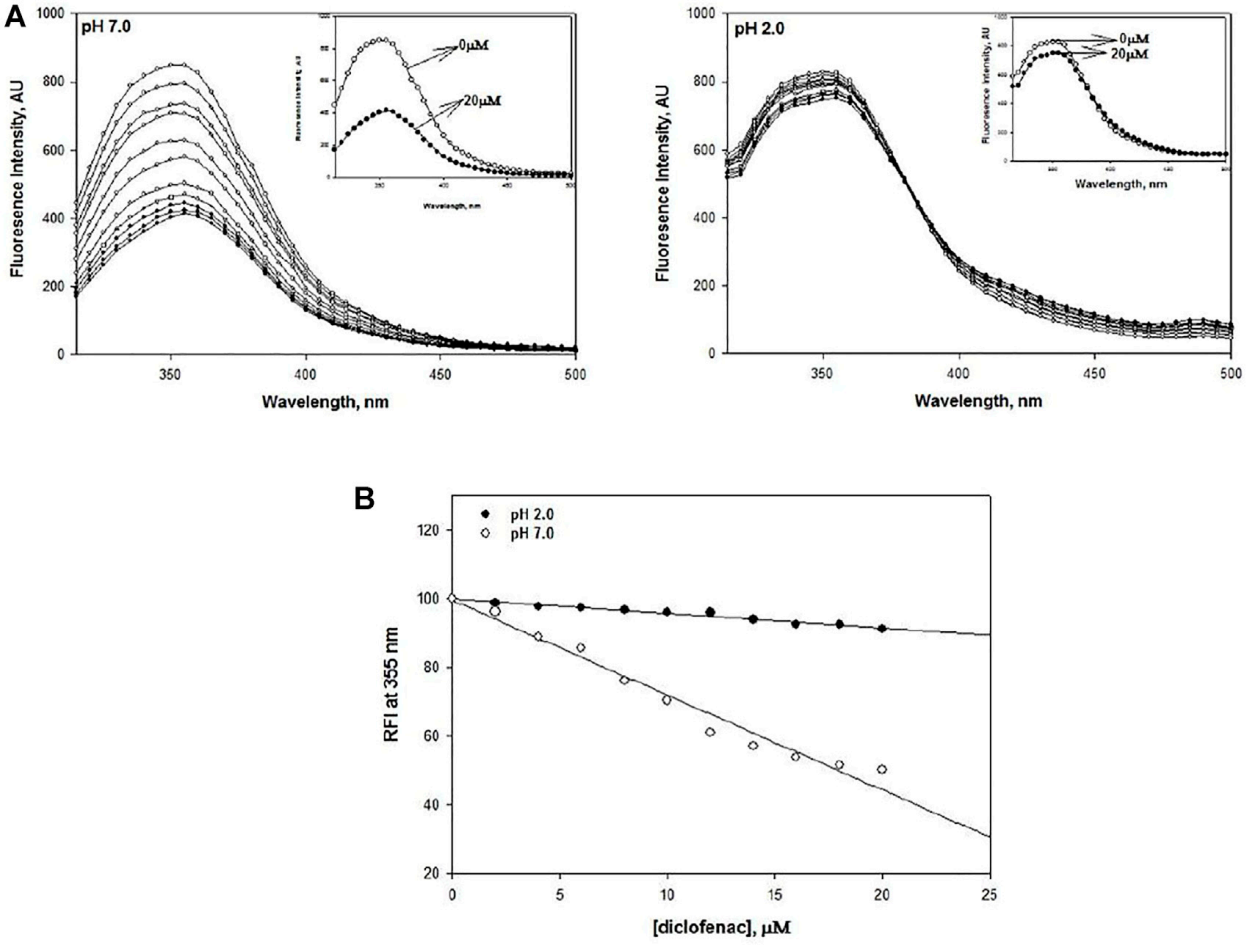
**(A)** DCF-induced fluorescence quenching of lysozyme at pH 2.0 and pH 7.0 at 25°C. The concentration of lysozyme was 20 μM. DCF concentrations varied from 2 to 20 μM in a successive increment of 2 μM. **(B)** Relative fluorescence intensity of DCF-induced quenching of lysozyme at pH 2.0 and 7.0.

To shed some light on the interaction of DCF with lysozyme, intrinsic fluorescence spectroscopy was carried out at pH 7.0 and pH 2.0. DCF concentrations in the range of 2–20 μM were used. The fluorescence emission spectra were recorded in the range of 315–500 nm with an excitation wavelength of 295 nm. The residues Trp 62 and Trp108 are the most dominant fluorophores present in this protein (Saha et al., 2018). Quenching was observed at both pH values, but the magnitude of quenching was less at pH 2.0 (Figure 4A and insets).

Earlier intrinsic fluorescence studies on various proteins have demonstrated that quenching of fluorescence intensity is an indicator of destabilization (Bansal et al., 2018). Hence, we can conclusively say that lysozyme gets destabilized in the presence of DCF. Our finding gets further support from the study conducted by Kenawi and coworkers, who implicated DCF’s ability to form hydrogen bonds and intermolecular charge transfer complex with proteins to be responsible for its destabilization (Kenawi et al., 2005; Bielecka et al., 2019; Paul et al., 2021). This phenomenon of quenching shows a linear decrease with an increase in DCF concentration. To validate this statement, relative fluorescence intensities (RFI) at 355 nm were plotted against DCF concentration at both pH values, which shows that there is a decrease in the RFI values with the addition of DCF (Figure 4B).

However, our finding leads us to speculate that the p*I* value of lysozyme and DCF are 11 and 4, respectively. Therefore, at pH 7.0, lysozyme exists as a positively charged structure, while DCF remains as a negative entity. This difference in like charge leads to the electrostatic attraction between both, thus bringing DCF in the close vicinity of our protein. This interaction of DCF with lysozyme leads to structural changes that cause a decrease in the stability of lysozyme. However, at pH 2.0, both lysozyme and DCF exist as positively charged entities. Hence, an electrostatic repulsive force exists between the two entities, which allows a small amount of DCF to bind to the lysozyme. This results in less extent of destabilization of lysozyme at acidic pH than at neutral pH.

The other way to explain the process of protein destabilization is through the contribution of enthalpy and entropy components, which play an important role in the thermodynamic stability. Enthalpy and entropy contribute to its stabilization in terms of Δ*G*_D_^o^, so Δ*H*_D_^o^ (Δ*H*_D_, the denaturation enthalpy change at 25°C) and Δ*S*_D_^o^ (Δ*S*_D_, the denaturation entropy change at 25°C) were calculated using the relations Δ*H*_D_^o^ = Δ*H*_m_ − Δ*C*_p_(Δ*T*_m_ − 298.15) and Δ*S*_D_^o^ = (Δ*H*_m_/*T*_m_) + Δ*C*_p_ ln(298.15/*T*_m_). The values of Δ*H*_D_^o^ and *T*Δ*S*_D_^o^ (where *T* is the temperature, in kelvin, at that specific DCF concentration) are given in Table 2.

**TABLE 2:** Change in stability parameters on transferring proteins from 0 to 20 μM DCF at different pH values.^a^

pH	[DCF], μM	Δ*H*_D_^o^	*T*Δ*S*_D_^o^
2.0	0	30.9	25.2
	5	33.3	27.9
	10	35.7	30.5
	15	37.1	32.5
	20	38.6	34.2
3.0	0	13.8	5.8
	5	17.7	9.6
	10	20.7	12.4
	15	23.0	14.8
	20	25.1	16.9
4.0	0	15.1	6.5
	5	19.4	10.6
	10	22.7	13.9
	15	25.1	16.3
	20	26.9	18.2
5.0	0	29.5	18.4
	5	33.8	22.7
	10	36.9	26.0
	15	38.6	27.8
	20	40.7	30.2
6.0	0	33.0	20.2
	5	37.2	24.6
	10	40.1	21.7
	15	42.5	30.6
	20	44.6	33.0
7.0	0	30.7	21.3
	5	35.0	25.8
	10	39.2	30.1
	15	42.0	34.6
	20	43.8	39.5

a Δ*H*_D_° is in kcal mol^−1^ and *T*Δ*S*_D_° is in kcal mol^−1^ *K* ^−1^.

To see whether the process of protein stabilization is enthalpically or entropically driven, values of ΔΔ*H*_D_^o^ versus *T*ΔΔ*S*_D_^o^ were calculated at pH 2.0 and 7.0 only (Table 3).

**TABLE 3:** Stability parameters of lysozyme in the presence of DCF at two pH values.

[DCF], μM	pH 2.0		pH 7.0	
	ΔΔ*H*_D_^o^	*T*ΔΔ*S*_D_^o^	ΔΔ*H*_D_^o^	*T*ΔΔ*S*_D_^o^
0.0	0	0	0	0
5.0	2.4	2.7	4.3	4.5
10.0	4.8	5.3	8.5	8.8
15.0	6.2	7.3	11.3	13.3
20.0	7.7	9.0	13.1	18.2

a ΔΔ*H*_D_° is in kcal mol^−1^ and *T*ΔΔ*S*_D_° is in kcal mol^−1^ *K* ^−1^.

**FIGURE 5:**
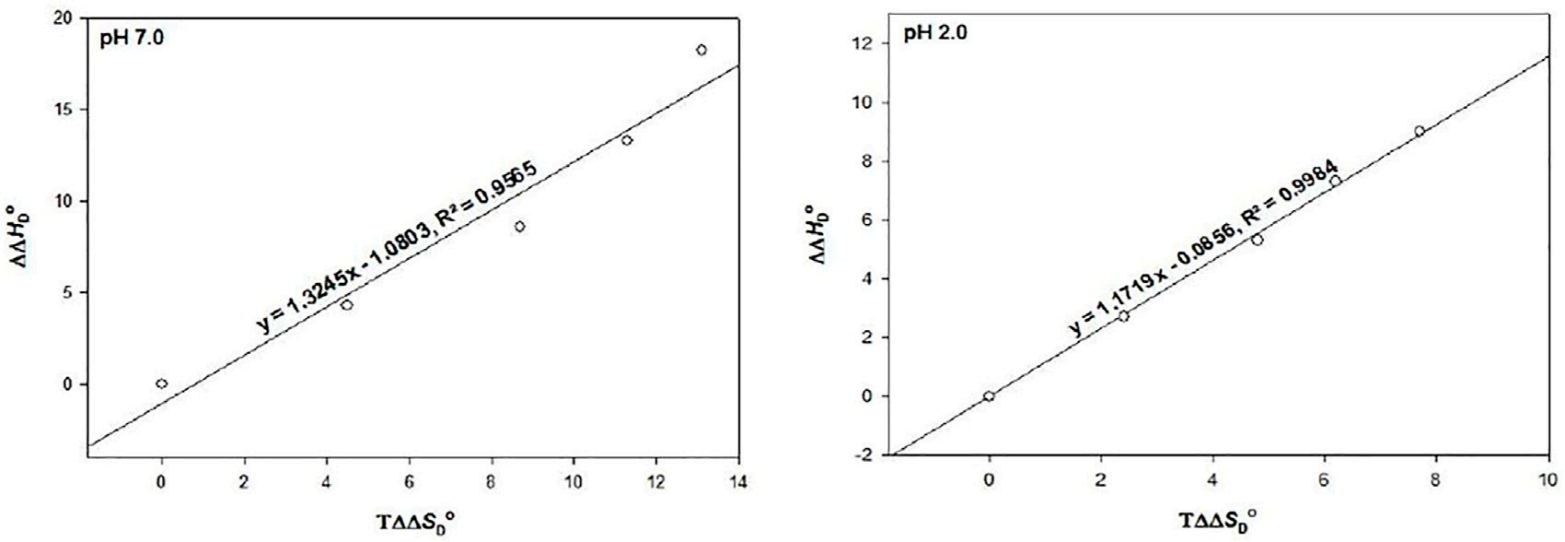
The plot of ∆∆*H*_D_^○^ versus *T*∆∆*S*_D_^○^ at pH 2.0 and pH 7.0. The values of each pH are those predicted from the results given in Table 3.

Using these values, a graph of ΔΔ*H*_D_^o^ versus *T*ΔΔ*S*_D_^o^ is plotted in the presence of different molar concentrations of DCF (Figure 5), which shows that there is no perfect enthalpy entropy compensation at both pH values. Rather, it can be seen that *T*ΔΔ*S*_D_^o^ > ΔΔ*H*_D_^o^; hence, the process of destabilization is entropically driven. The interaction between GdmCl and DCF was ruled out, as there is a linear trend found in a decrease in T_*m*_ in the presence of different concentrations of DCF with a fixed amount of GdmCl. Our results suggest that the entropic contribution to the protein destabilization overweighs the enthalpic contribution, which is further supported by the destabilizing effect of TMAO in RNase A, which also shows that this destabilizing effect is under entropic control. This finding is further supported by the destabilizing effect of RNase A in the presence of TMAO, which is also under entropically control (Singh et al., 2005).

The thermodynamic quantities are just the physical parameters that need to be validated with biological function. Henceforth, our observations were validated by measuring the kinetic parameters *K*_m_ and *k*_cat_ of lysozyme in the absence and presence of DCF at pH 7.0 (Table 4).

**TABLE 4:** Activity parameters of lysozyme in the absence and presence of DCF at pH 7.0 and 25°C.

[DCF], μM	*K*_m_ (μg ml^−1^)	*K*_cat_ (mg ml^−1^ s^−1^ M^−1^)	*K*_cat_/*K*_m_
0	77.8 ± 2	484.1 ± 29	6.22
5	86.7 ± 3	448.9 ± 21	5.16
10	92.5 ± 2	412.1 ± 24	4.45
15	95.3 ± 3	371.8 ± 26	3.90
20	104.8 ± 4	340.0 ± 27	3.22

It can be seen that with the addition of DCF, *K*_m_ of lysozyme increases, while *k*_cat_ is decreased. In the absence of DCF, the values of the enzymatic parameters of lysozyme agree with the earlier reports (Wetlaufer, 1963; Weir, 2002; Jamal et al., 2009; Khan et al., 2013; Antosiewicz and Shugar, 2016; Beltrán and Franco, 2019; Costa et al., 2019; Nambiar, 2019), and we assure that all the values obtained from this study are authentic and accurate. DCF destabilizes the lysozyme by shifting the denaturation equilibrium (native state ↔ denatured state) toward the right side because it has the ability to bind the enzyme (Langman et al., 1994; Grosser et al., 2006; Decherchi and Cavalli, 2020; Migliore et al., 2021). The above observation can be explained in the light of the change in the functionally native conformation of lysozyme at pH 7.0, and the presence of DCF leads to a change in the conformation of enzymes, making it inefficient/slow to complete the reaction. The change in the enzyme active site may be the subtle reason for the observation of *K*_m_ and *k*_cat_ values. This is in complete agreement with the previously published data on other proteins (Khan et al., 2013; Dragan et al., 2019). Since the overall catalytic activity of an enzyme cannot be defined by *k*_cat_ alone, the ratio of *k*_cat_ and *K*_m_ (*k*_cat_/*K*_m_) refers to the reaction of free enzyme and free substrate (Jamal et al., 2009; Nambiar, 2019), so the parameter *k*_cat_/*K*_m_ was calculated (Table 4). It can be seen that in the presence of DCF, the overall catalytic efficiency of lysozyme decreases. This effect shows that DCF affects the association, either through solvation effects on the substrate or enzyme active sites or their thermodynamic activities.

In our study, we found the destabilizing effect of DCF predominant at physiological pH, and this could be a reason that patients put on prolonged use of DCF have serious defects such as kidney damage and cardiovascular disorder. However, there is no direct evidence or reference. The hypothesis needs to be tested by conducting studies on a protein isolated from the heart, kidney, and stomach to understand the mechanism involved in the damage of these organs due to the DCF prolonged usage.

## Conclusion

Taken together, our outcomes suggest that DCF reduces the stability of protein at the physiological pH (pH 7.0). This decrease in the stability of the molecule is also reflected in the loss of structure at both the tertiary and secondary levels of its organization. The study on lysozyme also demonstrated a loss of function at the physiological pH in the presence of DCF. However, at a low pH, DCF exhibits no such effect on its structure, stability, and function.

## Data Availability

The original contributions presented in the study are included in the article/Supplementary Material. Further inquiries can be directed to the corresponding author.
